# Supplementary material for: Highly Thermal Conductive Graphite Films Derived from the Graphitization of Chemically Imidized Polyimide Films
Source: Nanomaterials (Basel). 2022 Jan 24;12(3):367. doi: 10.3390/nano12030367 (PMC8840353; doi:10.3390/nano12030367)
Supplement: Supplementary file 1 [file nanomaterials-12-00367-s001.zip › nanomaterials-1541420-supplementary.pdf]

nanomaterials

MDPI

Supporting Information

Highly Thermal Conductive Graphite Films Derived from the Graphitization of Chemically Imidized Polyimide Films

Meijiao Sun [1], Xiaoqiang Wang [1], Zhengyu Ye [1], Xiaodong Chen [2], Yuhua Xue [1] and Guangzhi Yang [1,*]

[1] School of Materials and Chemistry, University of Shanghai for Science and Technology, Shanghai 200093, China; 18307416856@163.com (M.S.); XQ320557@163.com (X.W.); 1935040329@st.usst.edu.cn (Z.Y.); xueyuehua@usst.edu.cn (Y.X.)
[2] Taihu Jinzhang Science & Technology (Anhui) Crop, Ltd., Anqing 246000, China; chenxd@jzt3.com
* Correspondence: yanggzh@usst.edu.cn; Tel.: +86-21-55270632

Table S1. The thickness and density of samples in the LFA measurement.

Sample	Thickness (μm)	Density (g·cm^{-1})
g-TIPI	9	1.68
g-CIPI-0.5%	10	1.85
g-CIPI-0.56%	10	1.73
g-CIPI-0.64%	9	1.74
g-CIPI-0.72%	9	2.10
g-CIPI-0.8%	8	2.12
